# Pooled incidence and predictors of infant mortality in low- and middle-income countries using gamma shared frailty model: Insights for achieving the Sustainable Development Goals

**DOI:** 10.1371/journal.pone.0347023

**Published:** 2026-04-16

**Authors:** Dejen Kahsay Asgedom, Habtamu Solomon Demeke, Etsay Woldu Anbesu, Ausman Ahmed Mohammed, Simachew Getaneh Endalamew, Bewuketu Terefe, Solomon Keflie Assefa, Asefa Asimasu Taddese

**Affiliations:** 1 Department of Public Health, College of Medicine and Health Science, Samara University, Samara, Ethiopia; 2 Department of Nursing, College of Medicine and Health Sciences, Samara University, Samara, Ethiopia; 3 Department of Veterinary Epidemiology and Public Health, School of Veterinary Medicine, Bahir Dar University, Bahir Dar, Ethiopia; 4 Department of Community Health Nursing, School of Nursing, College of Medicine and Health Science, University of Gondar, Gondar, Ethiopia; 5 Department of Public Health, Pawi Health Science College, Pawi, Ethiopia; 6 Academy of Wellness and Human Development, Faculty of Arts and Social Sciences, Hong Kong Baptist University, Hong Kong SAR, China; Flinders University, AUSTRALIA

## Abstract

**Background:**

Low- and middle-income countries (LMICs) account for a large share of global infant deaths, but there is a lack of evidence on the pooled estimate of infant mortality and its predictors in LMICs. Therefore, this study aimed to assess the pooled incidence of infant mortality and its associated factors in LMICs.

**Methods:**

We used clustered data extracted from the recent Demographic and Health Surveys (DHS 2018-DHS 2024) of all LMICs. A total of 1,404,826weighted numbers of recent live births were included in the study. A lognormal shared gamma frailty model was employed. We used the Akaike information criterion (AIC), Bayesian information criterion (BIC), and log-likelihood values for model comparison. An adjusted time ratio (ϕ) with a 95% confidence interval (CI) in the final model was used to select variables that had a significant association with time to infant death. The data were analyzed via R software version 4.3.1.

**Results:**

A total of 1,404,826 live births were included in the final analysis. By the end of the follow-up period, 72,569 infants (5.17%, 95% CI: 5.13–5.21) had died before their first birthday. The pooled estimate of the IMR in LMICs was 39 per 1000 live births (95% CI: 32.68–44.95). Maternal education, family size ≥ 5, being a multiparous mother, being delivered at health facilities, being a female infant, immediate initiation of breast feeding, living in Europe & Central Asia, and living in West & East Asia were significantly associated with a lower risk of infant death. Conversely, maternal age 25–34, maternal age 35–49, unimproved toilet facilities, poor and middle wealth indices, maternal age at birth ≤19, birth interval of <18 and 18–23 months, multiple births, 2^nd^ birth order, small birth size, low and medium Human Development Index (HDI), low and medium literacy rate, low-income and lower-middle income countries, rural residence, living in West Africa, South & Central Africa, and South Asia were significantly associated with a higher risk of infant mortality.

**Conclusion:**

The infant mortality rate (IMR) in LMICs remains high compared with that in WHO targets and shows significant regional variation. West Africa and South Asia had the highest pooled estimate of infant deaths. Variables such as maternal age, education, wealth index, age at first birth, parity, family size, child sex, birth interval, multiple pregnancy, birth order number, perceived child size at birth, place of delivery, residence, country’s literacy rate, income group, and HDI value were identified as significant predictors of time to infant death. Therefore, public health interventions that enhance health facility delivery, optimal birth spacing, maternal education, and immediate breastfeeding are crucial to reduce the incidence of infant mortality in LMICs.

## Introduction

The World Health Organization (WHO) defines infant mortality as the death of a child under one year of age—an immense, intolerable, and largely preventable loss of life [1].

Infant mortality remains a critical global challenge. According to the 2023 UNICEF report, an estimated 2.3 million children die in the first month of life, and 4.8 million children die before reaching their fifth birth day [2]. Although the global infant mortality rate has declined from 65 deaths per 1000 live births in 1990–27 in 2025 [3], it is the crucial population health metric that reflects a country’s capacity to provide health care and foster economic growth [4]. Despite this considerable progress, improving child survival remains a matter of urgent concern. In 2023 alone, 13,100 under-five deaths occurred every day, an intolerant high number of largely preventable child deaths [2].

Despite noteworthy progress at the global level, inequality across regions persists [3,4]. Children in Africa and Asia continue to have the highest risk of dying before they reach the age of five [5]. The United Nations Children’s Fund (UNICEF) and WHO data from 2023 indicate that infant mortality remains more prevalent in sub-Saharan Africa (SSA) (44 deaths per 1000 live births) and South Asia (32 deaths per 1000 live births) than in Australia and New Zealand (4 deaths per 1000 live births) [6–9]. The infant mortality risk LMICs exceeds that of high-income country by a factors of more than ten [2].

Risk factors for infant mortality include place of delivery [10–13], mode of delivery [11,13–15], antenatal care (ANC) during pregnancy [11,13,15], birth order [16], birth interval [10–13,15,17,18], child size and sex [4,19], maternal education [20], media exposure [21,22], and wealth level [23].

Despite progress, many low- and middle-income countries are not on track to achieve the Sustainable Development Goals (SDGs) target of reducing infant mortality to fewer than 25 deaths per 1,000 live births by 2030 [24]. At the current annual decline rate of 3.1%, the regional rate is projected to remain at 54 deaths per 1,000 live births by 2023, which is more than double the target. Without accelerating interventions, LMICs will fail to meet the SDG target [7]. A particular focus on intensifying efforts in LMICs is urgently needed [25].

LMICs bear a disproportionate burden of global infant mortality, yet comprehensive data on their pooled incidence and predictors across these nations are scarce. So far, there has been no pooled estimated of IMR in LMICs by controlling cluster-level frailty using Demographic and Health Survey Dataset (DHS). Furthermore, current comprehensive evidence on the pooled incidence of infant mortality will help policy makers and health programmers to monitor child survival trends and design targeted interventions. Understanding the factors influencing the time to infant death is crucial for improving child health and achieving the SDGs related to reducing child mortality in LMICs. Therefore, our objective was to determine the pooled incidence of infant mortality and its associated factors in LMICs.

## Methods and materials

### Study setting, design and period

This study utilizes nationally representative, cross-sectional data from 33 low- and middle-income countries (LMICs) between 2018 and 2024. The datasets were obtained from DHS Program website: https://www.dhsprogram.com, corresponding to the surveys conducted under the DHS-8 and DHS-9 phases. The individual country surveys were conducted between 2018 and 2024, based on the most recent available datasets for each country at the time of download. Countries with Standard DHS before 2018 were excluded due to the long-time data collection period since there has been demographic change over time. Following standard classification from the WHO and the DHS Program, the countries were grouped into six geographical subregions: East Africa, West Africa, South & Central Africa, South Asia, Europe & Central Asia, and West & East Asia. This extensive coverage enables a robust analysis of infant survival predictors across diverse LMICs.

### Source and study population

The source population comprised all live births in the five years preceding the survey across 33 LMICs. The study populations were all live births in the five years preceding the survey period in the selected enumeration areas (EAs) for each country.

### Sample size determination and sampling procedures

The DHS employs a two-stage sampling procedure that involves the selection of census enumeration areas (EAs) from each sampling stratum using a probability proportional to the size of the number of households in each EA in the first stage. In the second stage, households are sampled via systematic random sampling from each EA, which forms survey clusters [26]. To ensure national representativeness, we applied sample weights as recommended by the DHS Program. This adjustment corrects the oversampling and under-sampling inherent in complex survey design. The analysis was based on the most recent live births per mother. The data were extracted from the birth record (BR) file. A total of 1,404,826 weighted samples of the most recent live births were included in the analysis (Table 1 in S1 File).

### Study variables

#### Dependent variable.

The dependent variable was the time from the date of birth until the occurrence of death within the first year of life, measured in months. The outcome was coded as censored (0) or event (1).

#### Independent variables.

We considered the independent variables as follows: **socioeconomic factors** includes mothers age (15–24,25–34, 35–49) years, education (no education, primary, secondary, higher), current marital status (not married, married), current working (no, yes), partner’s education(no education, primary, secondary and above), partner’s working (no, yes), wealth index (poor, middle, rich), mass media exposure (no, yes), family size (1–4, ≥ 5), household source of drinking water (improved, unimproved), household type of toilet facility (improved, unimproved), country’s income group (upper middle income, lower middle income, low income), literacy rate (high, medium, low), Human Development Index (HDI) (High, medium, low), residence (urban, rural), and subregion (East Africa, West Africa, South & Central Africa, South Asia, Europe & Central Asia, West & East Asia), **obstetric and infant characteristics** includes mother’s age at first birth in years (≥20, ≤ 19), parity (primipara, multipara), number of ANC visit (zero, at least one), place of delivery (home, at health facility), delivered by cesarean-section (CS) (no, yes), sex of a child (male, female), twin (singleton, multiple), birth order (1^st^, 2^nd^, ≥ 3), preceding birth interval in months (≥24, 18–23, < 18), perceived size of the child at birth (larger than average, average, smaller than average), initiation of breast feeding (not immediate, immediate), child slept under mosquito net (no, yes), diarrhea within two weeks (no, yes), postnatal care within 2 months of birth (no, yes).

### Operational definition

**Event:** Infant deaths (1 = death).

**Censored:** Infants who were still alive and reached their first birthday at the end of the follow-up period were considered censored (0 = alive).

**The variable improved drinking water sources** include piped water, public taps, standpipes, tube wells, boreholes, protected dug wells, protected springs, and rainwater collection [27].

The predictor **media exposure** is a composite variable of frequency of listening radio, watching television and reading newspaper, in which households are said to have media exposure if they have been exposed to either listening radio or watching television or reading newspaper at least one week and have not been combined in to avoid exposure to all of the above media sources [28].

**The variable wealth index** was categorized as follows: the original categories “poorest” and “poorer” were combined into “poor”; “middle” remained unchanged; and “richest” and “richer” were combined into “rich” [28].

The variable **maternal educational level** was categorized as follows: the original categories “no education, incomplete primary, complete primary, incomplete secondary, complete secondary, and higher” were recoded to “no education, primary, secondary, and higher.”

The variable **infant size at birth** was categorized as follows: the original categories “very small” and “smaller than average” were combined into “smaller than average”; “average” remained unchanged; and “very large” and “larger than average” were combined into “larger than average.”

The variable **Human Development Index (HDI)** value is a composite statistic used to rank countries into four tiers of human development on the basis of long and healthy life (life expectancy), education (mean years of schooling and expected years of schooling), and standard of living (gross national income per capita). The HDI is used to categorize countries into four socioeconomic groups: very high, high, medium, and low HDIs [29]. Since there was no country in the very high HDI category, we categorized it into high, medium, and low HDIs.

**Country income level:** The country income variable was determined on the basis of the World Bank’s classification, which categorizes countries into low-income, lower-middle-income, and upper-middle-income groups [30]. A country is considered low income if its gross national income (GNI) per person was $1,135 or less in 2022. Countries with a GNI per capita falling between $1,136 and $4,465 are classified as having lower-middle income. The GNI per capita for upper-middle-income countries ranges from $4,466 to $13,845 [31].

**Country literacy rate:** According to data from the World Bank and World Population Review, developed countries typically have an average literacy rate above 90%, whereas the least developed nations have an average literacy rate of approximately 65% [32–34]. On this basis, we categorized countries’ literacy rates into three groups: high (countries with a literacy rate > 90%), medium (countries with a literacy rate between 75–90%) and low literacy rates (countries with a literacy rate below 75%).

**Geographic subregions:** The countries were classified according to the World Health Organization (WHO), DHS and UNICEF [33,35] into the following subregions: East Africa, West Africa, South & Central Africa, Europe & Central Asia, West & East Asia, and South Asia.

### Statistical analysis

To ensure cross-country comparability, we calculated the sampling weights using a denormalization approach and compared the results with those obtained using the original DHS weights. The sensitivity analysis showed no meaningful differences in the estimates. Therefore, for consistency with the standard DHS reporting practices, we retained the original weighted results in the final analysis.

Descriptive statistics are presented as weighted frequencies and percentages. To account for the unequal contribution of infants from different cohorts to the IMR denominator in the DHS data, country-specific IMRs and their standard errors were calculated via the DHS.rates R package. These estimates were then pooled via the meta-analysis function metagen() from the meta R package [36].

Prior to fitting the survival models, we assessed multicollinearity among covariates via the variance inflation factor (VIF). The results indicated no concerning collinearity, with a maximum variance inflation factor (VIF) of 3.39 for the human development index (HDI) variable and a mean variance inflation factor (VIF) of 1.5 across all covariates.

The Kaplan‒Meier failure curve was used to compare survival differences across variable categories. The log rank test was used to statistically compare survival between groups. The Schoenfeld residuals tests and graphical methods were used to check the Cox proportional hazard (PH) assumption before fitting the survival model for all possible predictors of time to infant mortality. The global Schoenfeld residual test indicated violation of the PH assumption (<<Eqn5>, suggesting that a Cox PH model would not be appropriate for this analysis.

Owing to the hierarchical nature of the DHS data, infants were nested within enumeration areas (EAs), which violates the assumption of independence. This implies the need to consider the between-clustering effect by using a mixed-effects model [37]. Therefore, a frailty model was chosen over traditional survival models. To account for the hierarchical structure of the data, where infants are nested within neighborhoods, we employed a frailty model (a random-effects survival model) to test for the presence of clustering/dependence.

In this context, frailty represents a cluster’s unmeasured risk. It is modeled as a random variable following a gamma distribution with a mean of 1 and an estimated variance (theta). The higher the frailty value is, the greater the degree of dependence.

To identify risk factors for infant mortality in LMICs, we fitted five parametric accelerated failure time (AFT) models: Weibull, exponential, log-normal, Gompertz, and log-logistic. We also compared gamma and inverse Gaussian shared frailty distributions. Model selection was based on the Akaike information criterion (AIC), Bayesian information criterion (BIC), and log-likelihood ratio (LLR) values.

The log-normal AFT model with gamma shared frailty demonstrated the best fit to our data. We first conducted bivariate analyses to assess individual variable associations. Variables showing an association with infant mortality at p≤0.25 in these initial analyses were included in the final multivariable log-normal AFT gamma shared frailty model. In the multivariable analysis, statistical significance was defined as p≤0.05.

In the final model, a significant theta value (θ=0.226, 95% CI: 0.204–0.25; p<0.001) indicated substantial unobserved heterogeneity, or shared frailty, among the clusters. This suggests that survival outcomes were more similar within the same cluster than between different EAs. We then used a likelihood ratio (LR) test to determine if the shared frailty model provided a better fit to the data than did a standard classical model. The significant result ($\chi^{\text{2}}(\text{1})=\text{1}\text{2}\text{7}\text{9}.\text{5}\text{6}$, p<0.001) confirmed that the frailty model was indeed superior. All analyses were performed via R software version 4.5.1.

We used complete case analyses for this study. The decision was made based on two factors: (1) the overall sample size remained sufficiently large even after excluding missing observations, preserving statistical power, and (2) our exploratory analyses suggested missingness was likely random, missing completely at random (MCA), primarily due to the recall issues inherent in DHS data collection.

To provide a comprehensive understanding of infant mortality, we employed both pooled meta-analysis and shared frailty survival analysis. The pooled meta-analysis provides a descriptive, macro-level estimate of infant mortality across the 33 LMICs, offering overall context and comparison. In contrast, the shared frailty survival analysis uses individual-level correlated DHS data to identify predictors of time to infant death, while accounting clustering and unobserved heterogeneity. Thus, the two approaches are complementary, addressing both the overall burden and determinants.

### Measure of dependence in the shared frailty model

For a Gamma shared frailty model, the level of dependence between infant survival times within the same cluster was quantified via Kendall’s tau (τ). This statistic measures the correlation between any two event times from the same cluster and is given by:

$$\begin{array}{c} \tau =\frac{\theta }{\theta +2} \end{array}$$

where θ represents the frailty variance and where τ ranges between 0 and 1. Higher values of θ indicate stronger within-cluster (EA) dependence, which correspondingly leads to higher values of Kendall’s tau (τ) [38].

### Ethical consideration

The analysis was based on secondary, deidentified data obtained from the DHS Program. To ensure ethical compliance, the authors adhered to the DHS Program protocol: all data were handled without access to personally identifiable information, which was removed prior to public release by the DHS Program. Therefore, ethical approval and participant consent were waived. Formal authorization for data use was secured from the DHS Program’s institutional review board prior to data download from https://www.dhsprogram.com.

## Results

### Characteristics of the study participants

This study included a weighted total of 1,404,826 reproductive-aged women with a live birth in the five years preceding the survey. The analysis revealed that nearly half, 628,309 (44.73%), of the mothers were between the ages of 25 and 34. The vast majority, 1,308,901 (93.23%), of the respondents were married. With respect to educational attainment, 557,917 (39.71%) mothers had no formal education. Nearly half of the households, 668,444 (47.58%), were in the poor category. Most households had access to improved drinking water sources (1,084,354 (77.19%) and improved toilet facilities 845,182 (60.16%). Geographically, the largest proportion of respondents were from South Asia (585,742; 41.69%) and West Africa (419,591; 29.87%).

The analysis revealed that the majority, 322,385 (91.97%), received at least one antenatal care visit during their pregnancy. Most deliveries, 375,087 (81.02%), took place in a health facility, and the fast majority, 1,062,172 (86.03%), were spontaneous vaginal deliveries. In terms of birth history, 754,998 (53.74%) of the women had their first birth at or before the age of 19. Among the infants, the sex distribution was balanced: 710,669 (50.59 male). The majority of children, 278,574 (61.52%), were reported to be of average size at birth. Just over half, 474,717 (51.50%) initiated breastfeeding immediately after birth. However, fewer than half of the 370,671 (39.43%) received a postnatal check-up within two weeks of delivery. The data revealed that 789,356 (56.19%) of the children were protected by a mosquito net. With respect to disease conditions, approximately 51,569 (10.46%) children had experienced a recent diarrheal episode (Table 1).

**Table 1:** Characteristics of the study participants in LMICs, 2018-2024.

A. Socioeconomic characteristics of the respondents				
Variables	Groups	Frequency		Weighted (%)
		Unweighted	Weighted	
**Mother’s current age**	15-24	182,461	181,452	12.92
	25-34	645,501	628,309	44.73
	35-49	630,540	595,065	42.36
**Education level**	No education	592,737	557,917	39.71
	Primary	341,732	324,173	23.08
	Secondary	432,884	424,710	30.23
	Higher	91,149	98,027	6.98
**Current marital status**	Not married	100,481	95,090	6.77
	Married	1,357,453	1,308,901	93.23
**Employment status**	No	435,596	407,633	43.31
	Yes	551,436	533,580	56.69
**Partner’s education**	No education	325,148	301,226	35.26
	Primary	203,432	192,113	22.49
	Secondary and above	370,018	361,032	42.26
**Partner’s working status**	Not working	72,317	63,902	7.52
	Working	821,133	786,123	92.48
**Wealth index**	Rich	412,282	455,412	32.42
	Middle	289,221	280,971	20.00
	Poor	756,999	668,444	47.58
**Mass media exposure**	No	524,982	486,456	34.63
	Yes	933,520	918,371	65.37
**Type of toilet facility**	Improved	860,609	845,182	60.16
	Unimproved	597,893	559,645	39.84
**Source of drinking water**	Improved	1,102,334	1,084,354	77.19
	Unimproved	356,168	320,472	22.81
**Country income group**	Upper Middle	108,094	98,935	7.04
	Lower Middle	1,058,390	1,020,776	72.66
	Low	292,018	285,116	20.30
**Country’s literacy rate**	High	73,023	66,993	4.77
	Medium	887,712	858,640	61.12
	Low	497,767	479,193	34.11
**HDI**	High	78,385	69,739	4.96
	Medium	959,371	936,003	66.63
	Low	420,746	399,084	28.41
**Place of residence**	Urban	414,249	449,044	31.96
	Rural	1,044,253	955,783	68.04
**Subregion**	East Africa	213,008	206,183	14.68
	West Africa	433,565	419,591	29.87
	South & Central Africa	116,671	108,280	7.71
	South Asia	602,992	585,742	41.69
	Europe & Central Asia	23,992	23,469	1.67
	East & West Asia	68,274	61,562	4.38
**B. Obstetric and infant characteristics**				
**Age at first birth**	≥ 20 years	680,717	649,828	46.26
	≤ 19 years	777,785	754,998	53.74
**Parity**	Prim para	703,950	685,212	48.78
	Multipara	754,552	719,615	51.22
**ANC visits**	Zero	30,674	28,132	8.03
	At least one	328,786	322,385	91.97
**Place of delivery**	Home	98,812	87,860	18.98
	Health facility	375,775	375,087	81.02
**PNC within 2 months**	No	600,225	569,508	60.57
	Yes	373,406	370,671	39.43
**Child delivered by C-section**	No	1,111,611	1,062,172	86.03
	Yes	164,839	172,540	13.97
**Sex of child**	Male	737,758	710,669	50.59
	Female	720,744	694,157	49.41
**Type of birth**	Single	1,424,124	1,371,645	97.64
	Multiple	34,378	33,181	2.36
**Birth order number**	1^st^	484,416	476,528	33.92
	2^nd^	365,792	357,984	25.48
	3rd and above	608,294	570,314	40.60
**Preceding birth interval**	≥ 24 months	696,852	664,260	71.86
	18-23 months	162,062	153,789	16.64
	<18 months	111,311	106,303	11.50
**Size at birth**	Larger than average	110,099	108,986	24.07
	Average	286,937	278,574	61.52
	Smaller than average	66,682	65,281	14.42
**Breastfeeding initiation**	Not immediate	457,785	447,002	48.50
	Immediate	505,321	474,717	51.50
**Child slept under Mosquito Net**	No	622,152	615,470	43.81
	Yes	836,350	789,356	56.19
**Diarrhea with 2 weeks**	No	454,891	441,645	89.54
	Yes	52,331	51,569	10.46
**Total**		**1,458,502**	**1,404,826**	**100**

### Pooled incidence of infant mortality in LMICs

**Fig 1:**
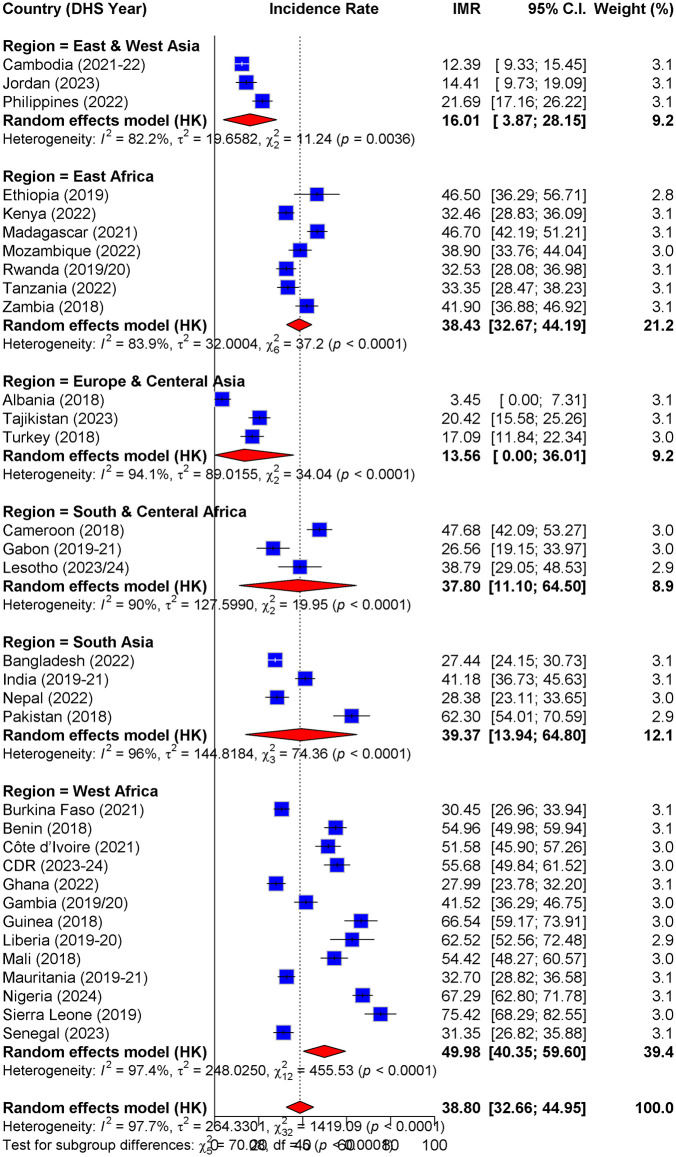
Pooled estimate of the IMR in low- and middle-income countries (2018 to 2024).

This study revealed a pooled infant mortality rate (IMR) of 39 per 1000 live births (95% CI: 32.66, 44.95) across 33 LMICs. A significant regional disparity was observed, with West Africa having the highest IMR (50 per 1000) and Europe & Central Asia the lowest (14 per 1000). At the country level, Sierra Leone had the highest IMR (75.45 per 1000), whereas Albania had the lowest (3.45 per 1000) (Fig 1).

**Fig 2:**
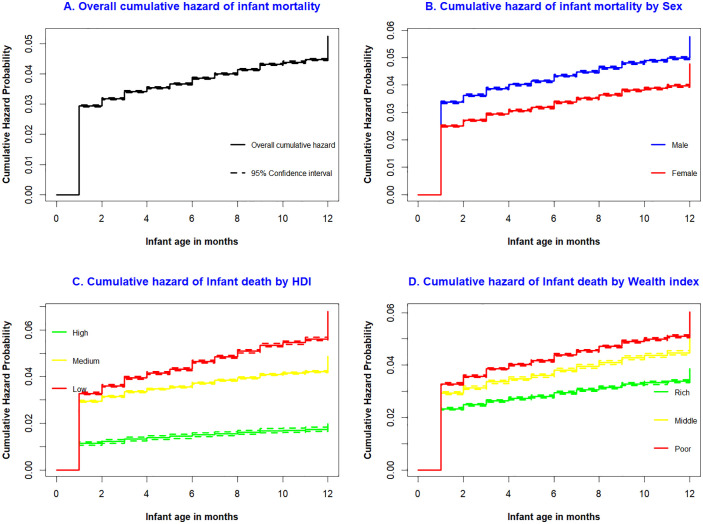
Overall cumulative hazard of infant mortality (Fig 2A), cumulative hazard of infant mortality by infant sex (Fig 2B), cumulative hazard of infant mortality by HDI (Fig 2C), and cumulative hazard of infant mortality by wealth index (Fig 2D).

The pooled infant mortality rate (IMR) in low-HDI countries was 51 per 1,000 live births (95% CI: 45.28–59.34), followed by medium-HDI countries (36 per 1,000; 95% CI: 28.29–43.04) and high-HDI countries (16 per 1,000; 95% CI: 5.59–29.19) (Fig 1 in S1 File). The pooled IMR in low-income countries 47 per 1,000 live births (95% CI: 34.56–60.30), followed by lower-middle income countries (39 per 1,000; 95% CI: 31.65–45.93) and upper-middle income countries (15 per 1,000; 95% CI: 0.00–44.27) (Fig 2 in S1 File).

### Hazard of infant death in LMICs

A total of 1,404,826 weighted live births were included in the final analysis. At the end of the follow-up period, 72,569 (5.17%, 95% CI: 5.13–5.21) had died before their first birthday. The cumulative hazard of death was estimated to be 3% by the end of the first month, 4% by the sixth month, and 5% by the end of the first year. Furthermore, variation by residence was observed; the cumulative probability of death at one year was 4% for infants in urban areas and 6% for those in rural areas (Table 2).

**Table 2:** Lifetime cumulative survival probability of infants in LMICs (2018-2024).

Time after birth	Cumulative Hazard Probability (95% CI)		
	Urban	Rural	Total
**Up to first month**	2.39% (2.35–2.44%)	3.16% (3.13–3.20%)	2.94% (2.92–2.97%)
**Third month**	2.74% (2.69–2.79%)	3.66% (3.62–3.70%)	3.40% (3.37–3.43%)
**Six months**	3.08% (3.03–3.13%)	4.14% (4.10–4.18%)	3.84% (3.81–3.87%)
**Nine months**	3.43% (3.38–3.49%)	4.60% (4.56–4.64%)	4.27% (4.23–4.30%)
**One year**	4.11% (4.05–4.17%)	5.59% (5.55–5.63%)	5.17% (5.14–5.21%)

### Kaplan–Meier failure curves

Nonparametric Kaplan–Meier failure curves were used to determine the probability of death of infants. The likelihood of mortality within the first month of life was high. Subsequently, beyond the initial month, the probability of survival for infants decreased proportionally (Fig 2A). Compared with their female counterparts, male infants were more likely to die (Fig 2B). Infants from countries with low-HDI had a greater cumulative hazard of death than infants from countries with medium and high-HDI did (Fig 2C). Compared with infants born to mothers in the rich wealth index category, those born to mothers in the poor and middle wealth indices had a greater cumulative hazard of death (Fig 2D).

### Factors affecting time to infant mortality in LMICs

To identify potential significant predictors of time to infant mortality, we employed a cluster-level parametric shared frailty survival model. The scale parameter in the lognormal AFT baseline hazard distribution gamma shared frailty model was determined to be σ = 3.52 (95% CI: 3.50, 3.55). This estimated scale parameter signifies that the hazard of infant death peaks early and then declines as age increases, reflecting an increasing survival rate.

Upon controlling for cluster-level frailty, the results from the final model indicated that variables such as maternal age, education, wealth index, family size, maternal age at first birth, parity, sex of child, birth interval, multiple pregnancy, birth order number, perceived child size at birth, place of delivery, residence, subregion country’s literacy rate, income group, and HDI value were identified as significant predictors of time to infant death.

Infant survival time was significantly associated with maternal age. Compared with infants born to mothers aged 15–25 years, those born to mothers aged 25–34 years had 27% shorter survival time (ϕ=0.73, 95% CI: 0.70, 0.76) and those born to mothers aged 35–49 years had 43% shorter survival time (ϕ = 0.57, 95% CI: 0.54, 0.60). Compared with infants born to mothers with no education, the adjusted survival time was 1.05 times longer for those whose mothers had primary education (ϕ = 1.05, 95% CI: 1.02, 1.09), 1.42 times longer for secondary education (ϕ= 1.42, 95% CI: 1.37, 1.47) education, and 2.24 times longer for those whose mothers completed higher education (ϕ = 2.24, 95% CI: 2.08, 2.42).

Infants from households in the middle wealth quantile had an 18% shorter survival time ϕ (= 0.82, 95% CI: 0.79, 0.85), and those from poor households had a 28% shorter survival time (ϕ = 0.72, 95% CI: 0.69, 0.75) compared to infants from the rich households. Infants from large families (≥ 5 members) had 71% longer survival time (ϕ = 1.71, 95% CI: 1.65, 1.77) compared with those from smaller families (1–4 members).

The survival time for infants born to households with unimproved toilet facilities was 8% shorter than those born to households with improved facilities (ϕ = 0.92, 95% CI: 0.89, 0.95).

The survival time of infants born to mothers who had their first birth at or before 19 was 40% decelerated than that of infants born to mothers who had their first birth after age 19 (ϕ = 0.60, 95% CI: 0.58, 0.61). Compared with infants born to first-time (primiparous) mothers, those born to multiparous mothers had a 61% longer survival time (ϕ = 1.61, 95% CI: 1.57, 1.65). Compared with those delivered at home, infants delivered at health facilities had a 92% longer survival rate (ϕ = 1.92, 95% CI: 1.84, 2.00).

Compared with male infants, female infants had a 45% longer survival time (ϕ = 1.45, 95% CI: 1.42, 1.49). Compared with those born at or after 24 months, infants born after a short birth interval of 18–23 months had a 65% shorter survival time (ϕ = 0.35, 95% CI: 0.34, 0.37). For very short intervals (< 18 months), the survival time was 85% shorter (ϕ = 0.14, 95% CI: 0.14, 0.15). Compared with first-born infants, second-born infants had a 9% decelerated survival rate (ϕ **=** 0.91, 95% CI: 0.87, 0.95). The survival time for multiple births was 95% shorter than singleton births (ϕ = 0.05, 95% CI: 0.05, 0.06). Infants perceived to be smaller than average at birth had a 60% shorter survival time (ϕ = 0.40, 95% CI: 0.37, 0.43) compared with those perceived to be large. The survival time of infants who were put to the breast immediately after birth was 7% longer than that of those who were not (95% CI: 1.04, 1.10).

The infant survival time in countries with a medium literacy rate is 29% shorter (ϕ=0.71, 95% CI:0.61,0.82), and in countries with a low literacy rate, it is 68% shorter lower (ϕ = 0.32, 95% CI: 0.27, 0.37) than the survival time in countries with a high literacy rate. Compared with infants in upper-middle income countries, those in lower-middle-income countries had a 56% shorter survival time (ϕ = 0.44, 95% CI: 0.35, 0.55), and those in low-income countries had a 64% shorter survival time (ϕ = 0.36 95% CI: 0.29, 0.46). The survival time for infants in medium-HDI countries was 66% shorter (ϕ= 0.34, 95% CI: 0.30, 0.38) than the survival time for infants in high-HDI countries. For infants from low-HDI countries, it was 63% shorter (ϕ= 0.37, 95% CI: 0.32, 0.42). Compared with those living in urban areas, those living in rural areas had a 20% shorter survival time (ϕ = 0.80, 95% CI: 0.77, 0.83). Finally, compared with East Africa, infant survival time was significantly longer in Europe & Central Asia (ϕ = 2.46, 95% CI: 1.95, 3.10) and East & West Asia (ϕ = 1.92, 95% CI: 1.67, 2.21) and significantly shorter in West Africa (ϕ = 0.67, 95% CI: 0.63, 0.71) and South & Central Africa (ϕ = 0.58, 95% CI: 0.55, 0.62) (Table 3).

**Table 3:** Factors associated with time to infant mortality in LMICs (2018-2024).

Variable	Categories	Coef (Std. Err.)	ϕ (95% CI)
**Maternal age**	(Ref: 15–24)		
	25-34	−0.315 (0.023)	0.73 (0.70, 0.76) ***
	35-49	−0.563 (0.025)	0.57 (0.54, 0.60) ***
**Maternal education**	(Ref: No education)		
	Primary	0.049 (0.017)	1.05 (1.02, 1.09) **
	Secondary	0.349 (0.019)	1.42 (1.37, 1.47) ***
	Higher	0.808 (0.039)	2.24 (2.08, 2.42) ***
**Wealth index**	(Ref: Rich)		
	Middle	−0.199 (0.021)	0.82 (0.79, 0.85) ***
	Poor	−0.334 (0.020)	0.72 (0.69, 0.75) ***
**Media exposure**	(Ref: No)		
	Yes	−0.005 (0.014)	0.99 (0.97, 1.02)
**Family size**	Ref: 1–4		
	≥ 5	0.53 (0.018)	1.71 (1.65, 1.77) ***
**House hold water source**	(Ref: Improved)		
	Unimproved	−0.020 (0.016)	0.98 (0.95, 1.01)
**Household toilet facility**	(Ref: Improved)		
	Unimproved	−0.082 (0.015)	0.92 (0.89, 0.95) ***
**Age at first birth**	(Ref: > 19)		
	≤19	−0.519 (0.014)	0.60 (0.58, 0.61) ***
**Parity**	(Ref: Primipara)		
	Multipara	0.476 (0.013)	1.61 (1.57, 1.65) ***
**Place of delivery**	(Ref: Home)		
	HF	0.651 (0.020)	1.92 (1.84, 2.00) ***
**Child sex**	(Ref: Male)		
	Female	0.375 (0.012)	1.45 (1.42, 1.49) ***
**Preceding birth interval**	(Ref: ≥ 24)		
	18-23	−1.038 (0.020)	0.35 (0.34, 0.37) ***
	<18	−1.955 (0.021)	0.14 (0.14, 0.15) ***
**Birth order number**	(Ref: 1st)		
	2nd	−0.097 (0.022)	0.91 (0.87, 0.95) ***
	3rd and above	0.006 (0.022)	0.99 (0.95, 1.03)
**Birth type**	(Ref: Single)		
	Multiple	−2.976 (0.029)	0.05 (0.05, 0.06) ***
**Infant’s size at birth**	(Ref: Larger)		
	Average	−0.057 (0.030)	0.94 (0.89, 1.00)
	Smaller than average	−0.921 (0.038)	0.40 (0.37, 0.43) ***
**When the child put to breast**	Ref: Not immediate		
	Immediate	0.066(0.014)	1.07 (1.04, 1.10) ***
**Infant slept under Net**	(Ref: No)		
	Yes	−0.012 (0.014)	0.99(0.96, 1.02)
**Country’s literacy rate**	(Ref: High)		
	Medium	−0.987 (0.075)	0.71 (0.61, 0.82) ***
	Low	−1.771 (0.809)	0.32 (0.27, 0.37) ***
**Country’s income group**	(Ref: Upper middle)		
	Lower middle	-.1.45 (0.121)	0.44 (0.35, 0.55) ***
	Low	−1.65 (0.122)	0.36 (0.29, 0.46) ***
**Country’s HDI**	(Ref: High)		
	Medium	−1.085 (0.066)	0.34 (0.30, 0.38)
	Low	−1.007 (0.066)	0.37 (0.32, 0.42)
**Residence**	(Ref: Urban)		
	Rural	−0.222 (0.018)	0.80 (0.77, 0.83)
**Geographic subregion**	(Ref: East Africa)		
	West Africa	−0.405 (0.029)	0.67 (0.63, 0.71)
	South & Central Africa	−0.537 (0.034)	0.58 (0.55, 0.62)
	South Asia	−0.190 (0.027)	0.83 (0.78, 0.87)
	Europe & Central Asia	0.899 (0.118)	2.46 (1.95, 3.10)
	East & West Asia	0.653 (0.071)	1.92 (1.67, 2.21)
σ = 3.52 (95% CI: 3.50, 3.55)			

***Coef*** coefficient of parameters, ***Std. Err.*** standard error of parameters, ϕ adjusted time ratio, ***95% CI*** 95% confidence interval for acceleration factor, σ scale parameter

### Random effect analysis and model comparison

After the information criteria were evaluated, it was determined that the lognormal baseline distribution model with a gamma frailty distribution provided the best fit. However, the lognormal baseline hazard distribution model, along with the inverse Gaussian frailty distribution model, failed to converge and did not yield meaningful results (Table 4).

**Table 4:** Model comparison and random effect analysis results.

Information criteria			Models	Frailty distributions
**LLR**	AIC	BIC		**Gamma**
−399,882.6	799,827.2	800,205.1	Exponential AFT	
−387,033.1	774,130.2	774,520.4	Gompertz	
−389,384.7	778,843.4	779,294.6	Weibull AFT	
−389,331.8	778,727.6	779,117.7	Loglogistic	
−386,972.1	774,008.1	774,398.3	Lognormal	
−400073.9	800219.9	800658.8	Exponential AFT	**Inverse Gaussian**
−387295.9	774665.8	775117.0	Gompertz	
−389352.9	778779.8	779231.0	Weibull AFT	
−388913.7	777901.3	778352.5	Loglogistic	
Not converged			Lognormal	
**Random effects results**				
**Parameters**		**estimates**	**95% CI**	
Theta (***θ***)		0.226	(0.204-0.25)	
Kendall’s tau (τ)		0.101	(0.09-0.113)	
LR test of theta = 0: chibar2 (01) = 1279.56 Prob ≥ chibar2 = 0.000				

The shared frailty model indicated significant heterogeneity in infant mortality between enumeration areas (EAs), with a frailty variance θ of 0.226(95%CI:0.205−0.25). A likelihood ratio test confirmed that this clustering effect was highly significant $\left(\chi^{\text{2}}(\text{1})=\text{1}\text{2}\text{7}\text{9}.\text{5}\text{6},p<\text{0}.\text{0}\text{0}\text{1}\right)$. The estimated within-cluster dependence, measured by Kendall’s τ, was 10.1%(95%,9.0−11.3). This finding indicates that approximately 10% of the total variation in infant mortality risk was attributable to unmeasured factors shared within EAs, equivalent to a correlation of approximately 0.10 in survival times among infants from the same cluster.

## Discussion

Reducing infant mortality is crucial for achieving the United Nations (UN) SDG 3 [39]. This study aimed to determine the pooled incidence of infant mortality and its associated factors in LMICs.

This study revealed a pooled infant mortality rate (IMR) of 39 per 1000 live births (95% CI: 32.66, 44.95) across 33 LMICs. The rate is substantially higher than those reported in high-income regions such as Australia and New Zealand (4 per 1000 live births) [8,19] and Europe (3.4 per 1000 live births) and exceeds the WHO target [39]. Significant regional disparities were observed, with West Africa having the highest IMR (50 per 1000) and Europe and Central Asia the lowest (14 per 1000). At the country level, Sierra Leone had the highest IMR (75.45 per 1000), whereas Albania had the lowest (3.45 per 1000). The highest incidence of infant mortality in LMICs might be linked to a combination of extreme poverty, deficient infrastructure, and low levels of education and health knowledge. Furthermore, environmental factors such as limited access to clean water have been identified as primary contributors to this crisis [40,41]. Additionally, the possible reason for the variation in IMR across regions and countries might be survey year differences. Another possible source of difference in the incidence of infant mortality across LMICs might be due to differences in universal healthcare coverage, socioeconomic context, and the adoption and implementation of policies and programs to reduce IMR.

This study revealed that infant survival was significantly associated with maternal age. Compared with infants born to mothers aged 15–25 years, the survival time was 27% shorter for those born to mothers aged 25–34 years and 43% shorter for those born to mothers aged 35–49 years. This finding is in line with the findings reported in studies conducted in Ethiopia [12] and Asia [42]. This evidence is also supported by a study conducted in California, USA [43], which revealed that younger and older maternal ages were associated with higher infant mortality. However, this finding is inconsistent with the findings of studies performed in East Africa [15]. Therefore, further study is recommended to address this inconsistency.

Higher maternal education was significantly associated with longer infant survival. Our findings are in line with the findings of studies conducted in Nepal [44], Nigeria [45], Nicaragua [46], Pakistan [47], Ethiopia [12,15], and East Africa [15]. A possible explanation is that education empowers mothers to adopt beneficial health practices and more effectively access quality care for their children [48].

We found that lower wealth was associated with shorter infant survival. Compared with infants from the richest households, the survival time was 18% shorter for those in the middle wealth category and 28% shorter for those in the poorest category. This finding is consistent with the findings of studies performed in SSA [49], Nepal [44], Bangladesh [50], and East Africa [15]. This disparity is likely driven by inequalities in access to critical resources. Wealthier families typically have better access to comprehensive healthcare services, including preventive check-ups, vaccinations, and emergency interventions. Furthermore, higher income facilitates a healthier living environment and more consistent access to nutritious food, both of which are fundamental to healthy child development [49,51,52].

Compared with those from smaller families (1–4 members), infants from large families (≥ 5 members) had a 71% longer survival time. This result supports one study in Ethiopia [12] but conflicts with others [11]. This inconsistency may stem from methodological differences, including the earlier study’s rural focus and the use of old data. Additionally, our family size variable included all household members, including visitors, during the data collection period, not just offspring. Given to the methodological differences and contextual variations, further longitudinal and context-specific studies are warranted studies are needed to compromise this inconsistency.

We found an 8% shorter infant survival time in households with unimproved toilet facilities compared with those in households with improved facilities, which aligns with other studies from SSA [53,54]. This is because the use of unimproved toilet facilities increases the risk of childhood acute respiratory infection and diarrhea, which are major causes of early childhood mortality and morbidity [53,55,56].

The survival time of infants born to mothers who had their first birth at or before 19 was 40% shorter than that of infants born to mothers who had their first birth after age 19, a finding that is consistent with the literature [13,57–60]. The underlying reasons for this association are multifactorial. Biologically, physical and physiological immaturity increases the likelihood of adverse outcomes such as inadequate prenatal weight gain and low birth weight [13,61]. Socially, younger mothers may have less decision-making power regarding infant care, as demonstrated by a qualitative study in South Africa [62]. Additionally, older pregnancy is considered a high-risk condition and is associated with a greater incidence of obstetric complications and adverse child outcomes [63,64]. These factors collectively explain the positive correlation between younger maternal age and infant mortality.

We found that infants born to multiparous mothers have a 61% longer survival time than do infants born to first-time (primiparous) mothers, which aligns with the findings of a previous Australian National Baboon Colony (ANBC) study [65]. This association can also be strongly explained by differences in maternal experience and infant care practices. The provided data indicate that primiparous mothers, who are often younger, face significant challenges that can compromise infant health. For example, they were more likely to abandon breastfeeding early (57.3% after 42 days) and reported more difficulties with breastfeeding both in the hospital and after discharge. Furthermore, primiparous mothers encountered more problems in caring for their newborns (79.7% vs. 65.6%) and were more inclined to use non-recommended remedies, such as teas for colic and substances on the umbilical cord, often on the basis of advice from relatives. In contrast, multiparous women demonstrated more established and potentially more effective caregiving routines. This collective evidence suggests that the survival advantage associated with multiparity is likely mediated by greater maternal experience, more successful breastfeeding practices, and more confident, evidence-based newborn care [65].

Our study revealed that infants delivered at health facilities had a 92% longer survival time than did those delivered at home. This finding is supported by numerous previous studies [10–13]. The primary explanation for this protective effect is immediate access to skilled birth attendants and modern medical equipment, which are crucial for managing obstetric complications.

This study revealed that female infants had a 45% longer survival time than male infants did, a finding supported by numerous previous studies [4,8,12,19–21,45,66]. This survival advantage among females is often attributed to genetic and biological variation. Male infants are generally more vulnerable to disease and premature death, whereas females have a biological advantage that supports survival in the first month of life [67]. Additionally, male fetuses are more likely to experience adverse conditions, such as intrauterine growth retardation, premature birth, and pregnancy-induced hypertension, all of which can contribute to early infant death.

We found that a short birth interval is significantly associated with a shorter infant survival time. Compared with those born at or after 24 months, infants born after a short birth interval of 18–23 months had a 65% shorter survival time. For very short intervals (< 18 months), the survival time was 85% shorter. This finding aligns with findings from other studies [10–13,15,17,18]. This is because short birth intervals are linked to a greater risk of adverse outcomes for the subsequent child, such as preterm birth and low birth weight IUCD [11,12]. Furthermore, a shorter interbirth interval is a significant risk factor for maternal depletion, competition between siblings for resources, and the transmission of infectious diseases [68]. These findings underscore the importance of the birth spacing recommendations from the World Health Organization (at least 2 years) [69].

This study also revealed that, compared with singleton births, twin higher-order births were associated with an increased risk of infant mortality, a finding that is consistent with studies from Ethiopia [11–13,15,17]. The increased risk can be attributed to a greater likelihood of obstetric complications such as obstructed labor and birth asphyxia. These factors increase susceptibility to respiratory infections and other complications that increase mortality risk in the first year of life [70,71]. This emphasizes the programmatic need for enhanced antenatal and intrapartum care for women with multiple pregnancies.

The present study revealed that infants perceived to be smaller than average at birth had a 60% shorter survival time. This finding is in line with the literature [8,11,45]. This is likely because infants perceived to be smaller than average at birth are more vulnerable to conditions such as neonatal sepsis, hypoglycemia and hypothermia [8,45].

We found that immediate breastfeeding after birth was associated with a 7% longer infant survival time, a finding that is consistent with previous studies [13,72–75]. This is because colostrum, the first milk, is rich in immunoglobulins and antibodies. These components help stimulate the newborn’s immune system and prevent gastrointestinal infections, thereby increasing survival [76,77].

Compared with those living in urban areas, those living in rural areas had a 20% shorter survival time. This finding is in line with findings from previous studies [78–80]. Research has shown that life expectancy and age are relatively short among rural residents [81]. The survival time for infants in medium-HDI countries is 66% shorter than that for infants in high-HDI countries. For infants from low-HDI countries, it was 63% shorter. This finding is consistent with findings from previous studies [82,83]. The human development index is one of the best indicators and predictors of perceived healthcare inequities. Improvements in these indicators worldwide, especially education level, might promote infant life expectancy and decrease infant mortality [83].

### Strengths and limitations of this study

- A key strength of this study is its application of a shared frailty modeling approach to control cluster-level dependency, thereby preventing misleading inferences and ensuring the valid interpretation of the results.
- By utilizing the most recent national representative datasets, the analysis offers a broad, comparative perspective on LMICs society and robustly identifies the predictors of time to infant mortality.
- Moreover, this study estimates the pooled incidence of infant mortality in LMICs.
- The use of secondary data restricted our ability to select and define exposure variables, potentially omitting other relevant factors from the analysis.
- The cross-sectional nature of the DHS data with retrospective histories may introduces inherent recall and survivor biases.
- Furthermore, despite the large sample complete case analysis (CCA) may introduce bias if missingness is related to unobserved factors and we recommend further study employ sensitivity analyses or multiple imputation (MI) to validate these findings.

## Conclusion

The infant mortality rate (IMR) in LMICs remains high compared with that in WHO targets and shows significant regional variation. West Africa and South Asia had the highest incidence of infant deaths. Variables such as maternal age, education, wealth index, age at first birth, parity, family size, child sex, birth interval, multiple pregnancy, birth order number, perceived child size at birth, place of delivery, residence, country’s literacy rate, income group, and HDI value were identified as significant predictors of time to infant death.

### Recommendations and policy implications

- To reduce infant mortality in LMICs, policy makers should prioritize enhancing health facility delivery, promoting optimal birth spacing, empowering women and expanding maternal education in the region.
- To reduce infant mortality in LMICs, policy makers and health stakeholders should pay special attention to modifiable risk factors such as immediate breastfeeding and teenage pregnancy.
- We also advise that region-specific strategies be developed for infants in West Africa, South Asia, and low-income and low-HDI countries.
- Targeted initiatives aimed at addressing socioeconomic disparities, such as wealth inequality and family size, can also play a pivotal role in reducing IMR in LMICs.

## Supporting information

S1 File Table 1. Sample size for pooled incidence and predictors of infant mortality in LMICs, 2018-2024. Fig 1. Forest plot of the pooled estimate of IMR by country HDI value across LMICs using the recent DHS between 2015 to 2024. Fig 2. Forest plot of the pooled estimate of IMR by income group across LMICs using the recent DHS between 2018 to 2024. (DOCX)

## Abbreviations

**AIC**: Akaike information criterion
**ANC**: antenatal care
**BIC**: Bayesian information criterion
**CI**: confidence interval
DHS: demographic and health survey
HDI: human development index
**IMR**: infant mortality rate
**KM**: Kaplan–Meier
**LLR**: log-likelihood ratio
**LMICs**: low- and middle-income countries
PH: proportional hazard
**SDGs**: sustainable development goals
SSA: sub-Saharan Africa
**WHO**: World Health Organization
