# Supplementary material for: Pooled incidence and predictors of infant mortality in low- and middle-income countries using gamma shared frailty model: Insights for achieving the Sustainable Development Goals
Source: PLoS One. 2026 Apr 16;21(4):e0347023. doi: 10.1371/journal.pone.0347023 (PMC13086360; doi:10.1371/journal.pone.0347023)
Supplement: S1 File — (DOCX) [file pone.0347023.s001.docx]

**Supporting information**

**Table 1. Sample size for pooled incidence and predictors of infant mortality in LMICs, 2018-2024.**

| **Country (DHS year)** | **Weighted sample** | | | **Unweighted samples** | | |
| --- | --- | --- | --- | --- | --- | --- |
|  | **Survival status** | | | **Survival status** | | |
|  | **Censored** | **Event** | **Total** | **Censored** | **Event** | **Total** |
| Albania (2018) | 5,006 | 32 | 5,038 | 5,443 | 39 | 5,482 |
| Bangladesh (2022) | 29,678 | 1,307 | 30,985 | 29,459 | 1,338 | 30,797 |
| Burkina Faso (2021) | 37,720 | 2,163 | 39,884 | 37,990 | 2,224 | 40,214 |
| Benin (2018) | 35,019 | 2,827 | 37,846 | 35,099 | 2,862 | 37,961 |
| Cameroon (2018) | 25,599 | 1,914 | 27,513 | 24,717 | 1,832 | 26,549 |
| Côte d’Ivoire (2021) | 25,976 | 1,758 | 27,734 | 29,506 | 2,314 | 31,820 |
| Cambodia (2021-22) | 17,625 | 414 | 18,039 | 18,743 | 500 | 19,243 |
| CDR (2023-24) | 56,738 | 3,382 | 60,120 | 62,703 | 4,019 | 66,722 |
| Ethiopia (2019) | 15,263 | 1,084 | 16,347 | 15,335 | 1,036 | 16,371 |
| Gabon (2019-21) | 15,107 | 439 | 15,546 | 17,054 | 580 | 17,634 |
| Ghana (2022) | 22,506 | 876 | 23,382 | 24,702 | 975 | 25,677 |
| Gambia (2019/20) | 24,008 | 1,425 | 25,433 | 26,488 | 1,684 | 28,172 |
| Guinea (2018) | 21,578 | 1,852 | 23,430 | 22,100 | 1,916 | 24,016 |
| India (2019-21) | 493,561 | 21,588 | 515,149 | 503,280 | 21,614 | 524,894 |
| Jordan (2023) | 21,431 | 282 | 21,713 | 24,637 | 277 | 24,914 |
| Kenya (2022) | 44,222 | 1,807 | 46,029 | 50,798 | 1,985 | 52,783 |
| Lesotho (2023/24) | 4,868 | 233 | 5,101 | 5,492 | 274 | 5,766 |
| Liberia (2019-20) | 14,288 | 1,506 | 15,794 | 16,522 | 1,838 | 18,360 |
| Madagascar (2021) | 31,838 | 1,726 | 33,564 | 32,640 | 1,765 | 34,405 |
| Mali (2018) | 27,671 | 2,586 | 30,257 | 25,914 | 2,300 | 28,214 |
| Mauritania (2019-21) | 33,045 | 1,357 | 34,401 | 32,750 | 1,426 | 34,176 |
| Mozambique (2022) | 25,763 | 1,268 | 27,030 | 24,837 | 1,370 | 26,207 |
| Nigeria (2024) | 90,628 | 8,829 | 99,457 | 90,570 | 8,694 | 99,264 |
| Nepal (2022) | 11,625 | 575 | 12,200 | 12,212 | 676 | 12,888 |
| Pakistan (2018) | 25,519 | 1,888 | 27,408 | 32,303 | 2,110 | 34,413 |
| Philippines (2022) | 21,387 | 424 | 21,811 | 23,618 | 499 | 24,117 |
| Rwanda (2019/20) | 21,390 | 1,153 | 22,543 | 20,947 | 1,113 | 22,060 |
| Sierra Leone (2019) | 27,213 | 3,167 | 30,380 | 28,049 | 3,120 | 31,169 |
| Senegal (2023) | 30,333 | 1,259 | 31,592 | 32,989 | 1,533 | 34,522 |
| Tajikistan (2023) | 12,494 | 306 | 12,800 | 11,981 | 291 | 12,272 |
| Turkey (2018) | 5,508 | 123 | 5,631 | 6,095 | 143 | 6,238 |
| Tanzania (2022) | 29,386 | 1,341 | 30,727 | 29,353 | 1,330 | 30,683 |
| Zambia (2018) | 28,264 | 1,679 | 29,942 | 28,769 | 1,730 | 30,499 |
| **Grand total** | **1,332,257** | **72,569** | **1,404,826** | **1,383,095** | **75,407** | **1,458,502** |

**
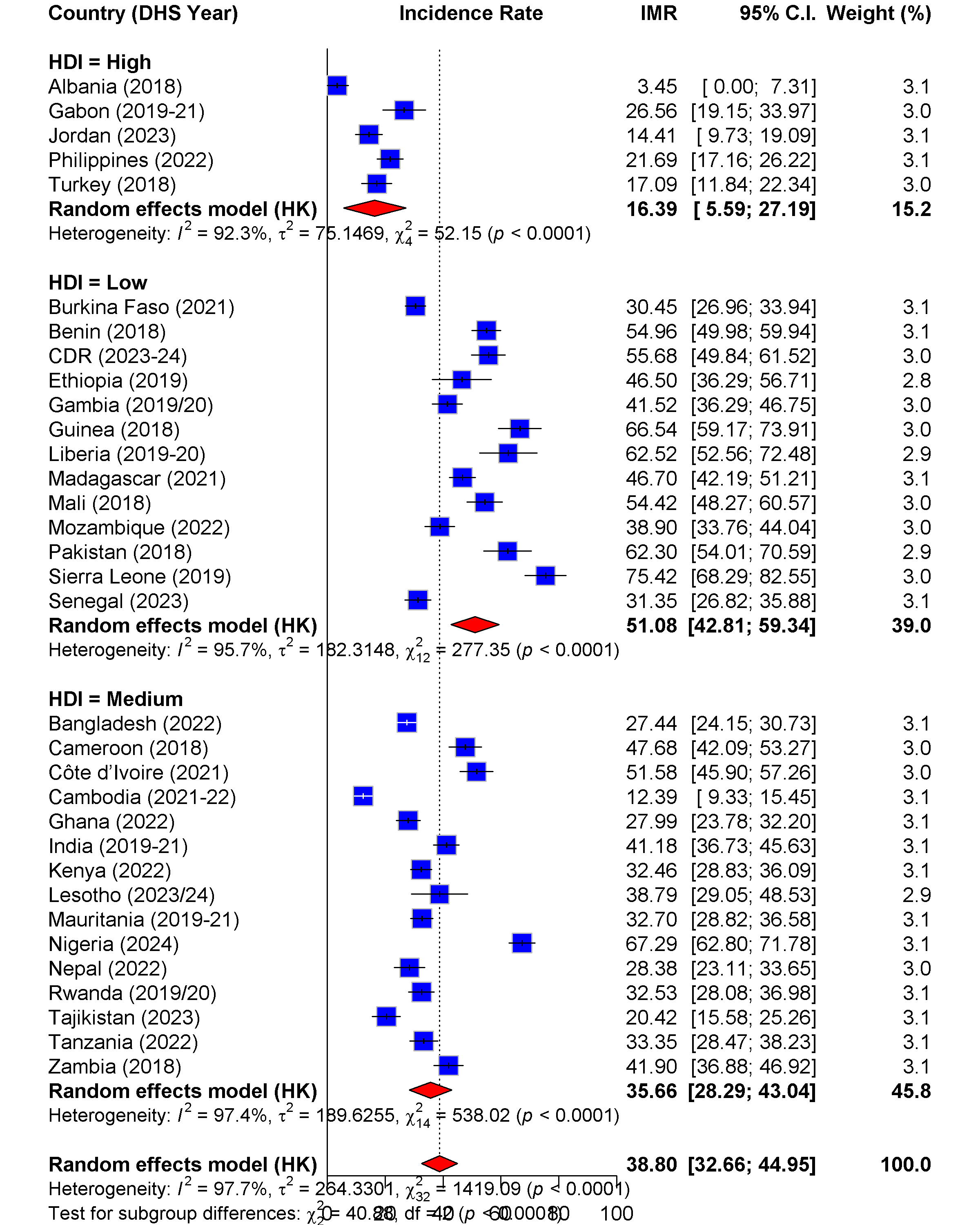
**

**Fig 1. Forest plot of the pooled estimate of IMR by country HDI value across LMICs using the recent DHS between 2015 to 2024**

**
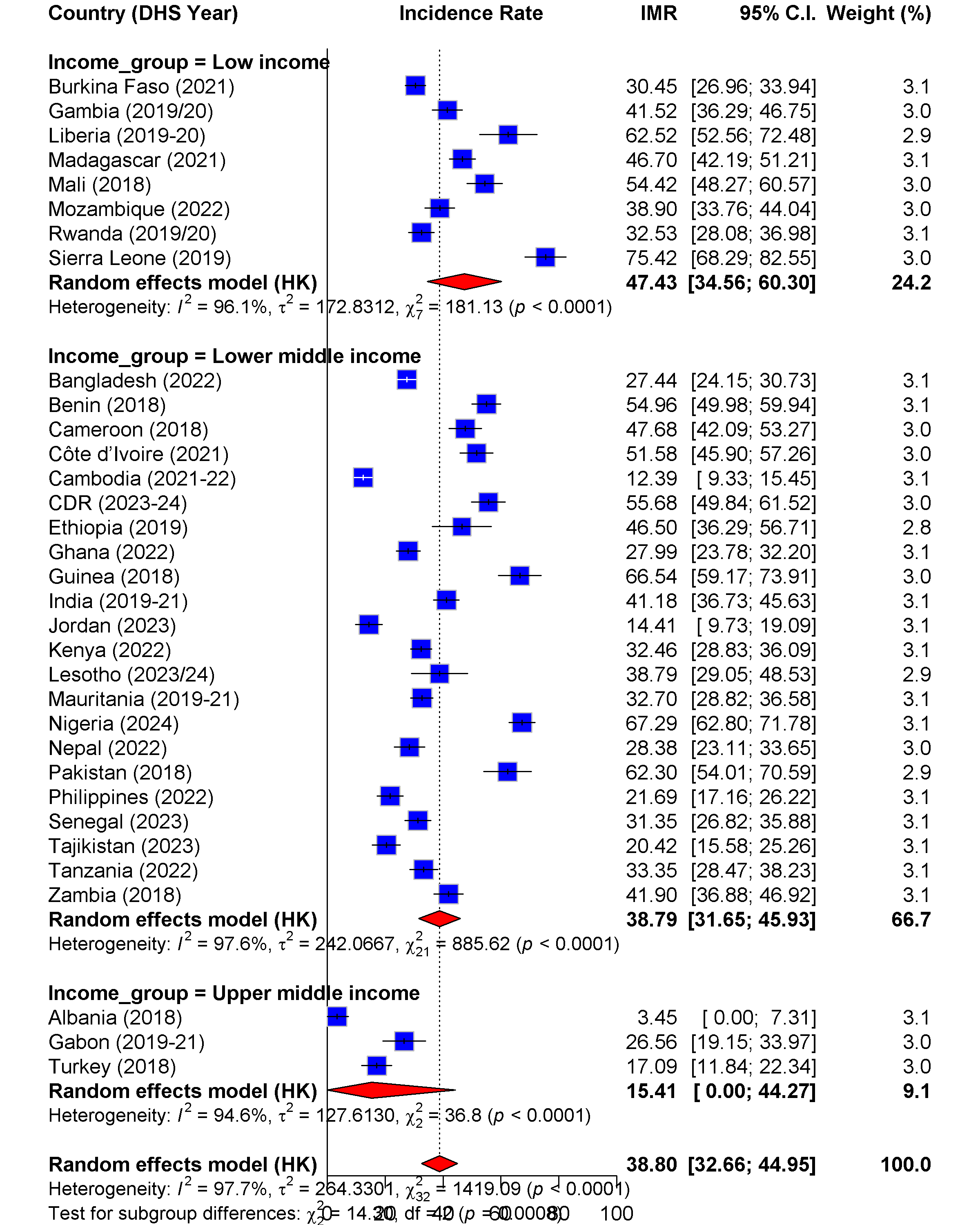
**

**Fig 2. Forest plot of the pooled estimate of IMR by income group across LMICs using the recent DHS between 2018 to 2024.**
